# Supplementary material for: Complexome profiling of the Chlamydomonas psb28 mutant reveals TEF5 as an early PSII assembly factor
Source: Plant Cell. 2025 Mar 17;37(6):koaf055. doi: 10.1093/plcell/koaf055 (PMC12164590; doi:10.1093/plcell/koaf055)
Supplement: koaf055_Supplementary_Data [file koaf055_supplementary_data.zip › psb28_interactive.zip!/plots/intensity/qualityPlots/peptides/USP_Cre14.g621751.t1.1.html]

### Cre14.g621751.t1.1

Trivial name: USP  
SeqCoverage [median]: 9.10  
Mass: 89.11 kDa  
  
 

| Sequence | MissedCleavage | Mass [Da] | wtIntensity | muIntensity | Score |
| --- | --- | --- | --- | --- | --- |
| HGYDMNSLAVPR | 0 | 1358.64 | 599.02 | 621.20 | 60.49 |
| KLPLAIMTSDDTHGR | 1 | 1653.85 | 0.00 | 739.83 | 41.54 |
| LGYSGIK | 0 | 736.41 | 516.68 | 0.00 | 87.28 |
| LSRPAGPEPK | 1 | 1050.58 | 3885.81 | 4960.88 | 67.73 |
| SNAALLSDKER | 1 | 1202.63 | 2704.85 | 5125.31 | 88.19 |
| VVLGPAFAPSLADIK | 0 | 1496.86 | 747.31 | 0.00 | 93.26 |
|  |  |  | 8453.7 | 11447.2 |  |
